# Bacteriophage DW-EC with the capability to destruct and inhibit biofilm formed by several pathogenic bacteria

**DOI:** 10.1038/s41598-022-22042-1

**Published:** 2022-11-03

**Authors:** Olivia Dwijayanti Wiguna, Diana Elizabeth Waturangi, Yogiara

**Affiliations:** Master of Biotechnology Department, Faculty of Biotechnology, Atma Jaya Catholic University of Indonesia, Jenderal Sudirman 51 Street, South Jakarta, DKI Jakarta, 12930 Indonesia

**Keywords:** Biotechnology, Microbiology

## Abstract

Biofilm formation by pathogenic bacteria is a major challenge in the food industry. Once a biofilm is established, such as on food processing equipment, it becomes more difficult to eradicate. Although physical and chemical treatments are often used to control biofilm formation, these treatments can have significant drawbacks. Alternative biofilm treatments are needed. Phage DW-EC was isolated from *dawet*, an Indonesian traditional Ready-To-Eat food, which has high specificity for Enterohaemorrhagic *Escherichia coli* (EHEC), Enteropathogenic *E. coli* (EPEC), and Enterotoxigenic *E. coli* (ETEC). Phage DW-EC produces several enzymes that can prevent the development of biofilm and biofilm eradication. Depolymerase enzymes break down the polysaccharides layer on the biofilms can lead to biofilm damage. On the other hand, endolysin and putative like-T4 lysozyme will lyse and kill a bacterial cell, thereby preventing biofilm growth. This research aims to determine the capability of previously identified phage DW-EC to inhibit and destroy biofilms produced by several foodborne pathogens. Phage DW-EC formed plaques on the bacterial lawns of EHEC, EPEC, and ETEC. The efficiency of plating (EOP) values for EHEC, EPEC, ETEC, and *Bacillus cereus* were 1.06, 0.78. 0.70, and 0.00, demonstrating that DW-EC was effective in controlling pathogenic *E. coli* populations. Furthermore, phage DW-EC showed anti-biofilm activity against foodborne pathogenic bacteria on polystyrene and stainless-steel substrates. DW-EC biofilm inhibition and destruction activities against pathogenic *E. coli* were significantly higher than against *B. cereus* biofilms, which was indicated by a lower density of the biofilm than *B. cereus.* Microscopic visualization verified that bacteriophage DW-EC effectively controlled EHEC, EPEC, and ETEC biofilms. The results showed that DW-EC could inhibit and destroy biofilm, making it promising to be used as an anti-biofilm candidate for polystyrene and stainless steel equipment in the food industry.

## Introduction

Food is the main transmission route of foodborne pathogens that can cause disease. These pathogens can cause infections, poisoning, and even death, posing a threat to public health. The most common foodborne pathogens, which include *Bacillus cereus* and *Escherichia coli*, can form biofilms on various matrixes, including food and industrial food processing instruments. Biofilms can be a source of pathogen contamination of processing equipment and food products, which can ultimately impact food product shelf life by accelerating the deterioration and spoilage of food. This shortening of shelf life ultimately results in economic losses due to consumer rejection of these food products. Moreover, contamination by foodborne pathogenic bacteria is a major consumer safety issue. Biofilms provide bacteria protection against antimicrobial substances as well as sanitizing agents, making the pathogenic bacteria more difficult to eradicate^1,2^.

The common strategy to eradicate biofilms in food industry equipment is using either physical treatments or chemical agents. However, this technique might harm product quality, negatively impact the environment, or damage or leave residue on equipment^2^. Therefore, an alternative strategy is needed to control biofilm formation by foodborne pathogenic bacteria.

Bacteriophages are abundant, naturally occurring viruses that can infect specific bacteria. Phages offer the advantages of specificity, self-replication, and non-toxicity. Lytic bacteriophages are often used to control foodborne pathogens and biofilms, and their use has been recognized as safe by the U.S. Food and Drug Administration^3^.

One such bacteriophage is DW-EC, previously isolated from *dawet*, a traditional Indonesian ready-to-eat food. Phage DW-EC was reported for its high specificity against several pathogenic *E. coli*, especially EHEC, EPEC, and ETEC. Bacteriophage DW-EC was applied to several foods such as chicken meat, fish meat, lettuce, cucumber, tomato, and pasteurized milk showed the ability to lyse and reduce ETEC significantly^4^. Phage DW-EC showed significantly reduced bacteria EHEC and ETEC on artificially contaminated dawet^5^. According to molecular analysis, phage DW-EC is a lytic phage because it does not encode the integrase dan excisionase gene. Besides that, DW-EC also does not have antimicrobial resistance (ARG) genes present in the genome. Bacteriophage DW-EC produces endolysin and putative like-T4 lysozyme can inhibit biofilm formation by lysing and killing each cell to prevent biofilm formation. On the other hand, depolymerase enzymes are also produced, which can degrade the extracellular polymeric substance (EPS) layer, so that, bacteriophages can lead to biofilm disruption and damage^4^.

Based on the previous research, bacteriophage DW-EC can be used as an alternative approach to reducing or controlling pathogenic bacteria. Phage DW-EC is a lytic phage that lyses host cells and does not integrate into the host genome, so that, horizontal gene transfer may be minimized. Further, those phages also produce enzymes that can be used to control and reduce biofilms. Therefore, this research is a continuation of the previous one^4^. This study aims to determine the ability of DW-EC to disrupt and inhibit biofilms of several pathogenic foodborne bacteria. Phage-encoded enzymes are expected to target the process of microcolony formation and biofilm maturation during the biofilm formation stage in several surface, such as polystyrene and stainless steel.

Based on the characteristics of DW-EC, it can be concluded that research to determine the ability of phage DW-EC as an anti-biofilm is needed, so that, it can be an alternative for biofilm treatment in food processing equipment in the food industry. The use of DW-EC can be a more eco-friendly and safer method compared use the chemical agent. In addition, the findings of this study may be a reference for further research on the ability of DW-EC as an anti-biofilm, wherein the phage-encoded enzyme can be purified and become an alternative as an anti-biofilm agent.

## Results

### Bacteriophage titer determination

Bacteriophage DW-EC was obtained from previous research in which it was isolated from dawet^4^. The results of the agar overlay assay indicated the presence of bacteriophage plaques. The phage DW-EC titer was shown to be approximately 1.47 ± 4.41 × 10^7^ PFU/mL.

### Host spectrum determination and efficiency of plating (EOP)

The host range spectrum was determined to identify the ability of phage DW-EC to infect pathogenic bacteria besides the host bacteria. We found that phage DW-EC could infect EHEC, EPEC, and ETEC but not *B. cereus*. Phage DW-EC was found to be highly effective against EHEC and EPEC, with an EOP of more than 0.70 (Table 1).

**Table 1:** Host spectrum and efficiency of plating (EOP) of bacteriophage DW-EC.

Bacteriophage isolate	Target bacteria			
	*B. cereus*	EHEC	EPEC	ETEC
DW- EC	0.00 ± 0.00^a^	1.06 ± 2.08^c^	0.78 ± 2.40^b^	0.70 ± 1.45^b^

Different letters indicate significant different between DW-EC treatment to against several biofilm pathogenic bacteria with *P* ≤ 0.05.

### Antibiofilm activity of DW-EC on polystyrene

**Figure 1:**
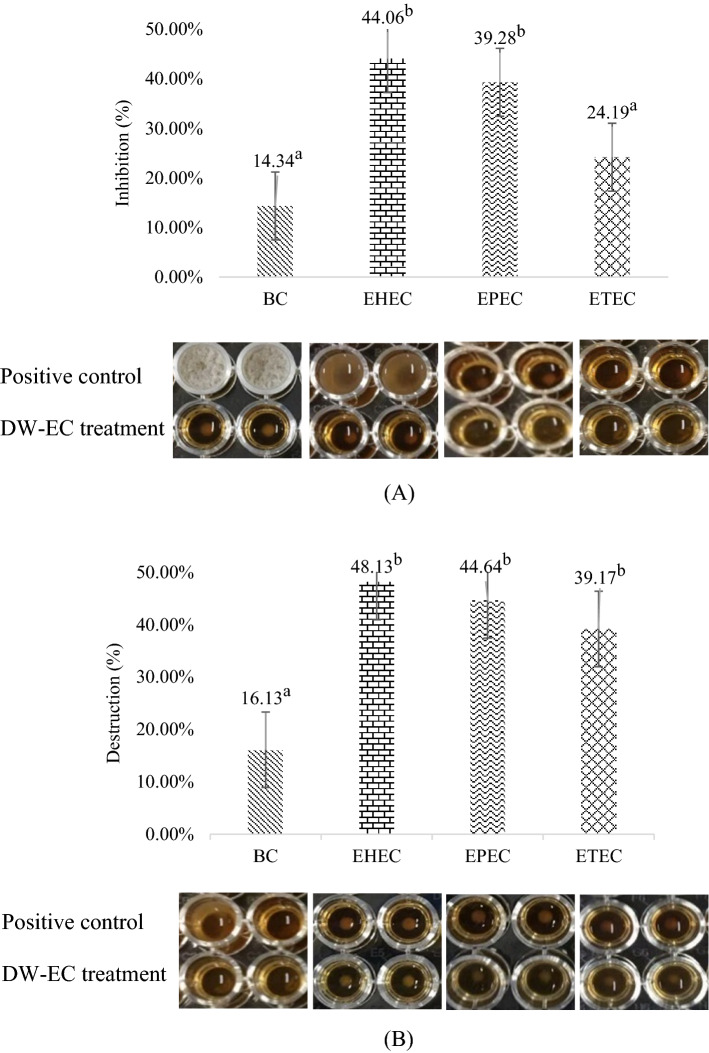
Application of bacteriophage DW-EC against pathogenic bacteria on polystyrene: (**A**) inhibition and (**B**) destruction activity. Different letters indicate significant different between DW-EC treatment to against several biofilm pathogenic bacteria with *P* ≤ 0.05.

A biofilm assay was used to determine the ability of bacteriophage DW-EC to inhibit (0-day old biofilm) and disrupt (1-day old biofilm) pathogenic bacterial biofilms. Bacteriophage DW-EC inhibited and destroyed EHEC, EPEC, and ETEC biofilms formed on polystyrene. Moreover, phage DW-EC showed higher destructive than inhibition activity. The highest inhibitory and destructive activity, 44.06% and 48.13%, respectively, was found against EHEC biofilms (Fig. 1), followed by EPEC, ETEC, and *B. cereus*.

### Antibiofilm activity of DW-EC on stainless-steel coupon

**Figure 2:**
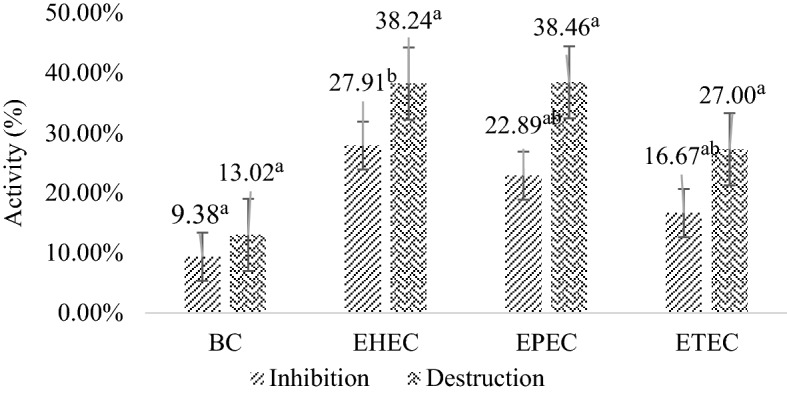
Inhibition and destruction activity of bacteriophage DW-EC against pathogenic bacteria on stainless-steel. Different letters indicate significant different between DW-EC treatment to against several biofilm pathogenic bacteria with *P* ≤ 0.05.

**Figure 3:**
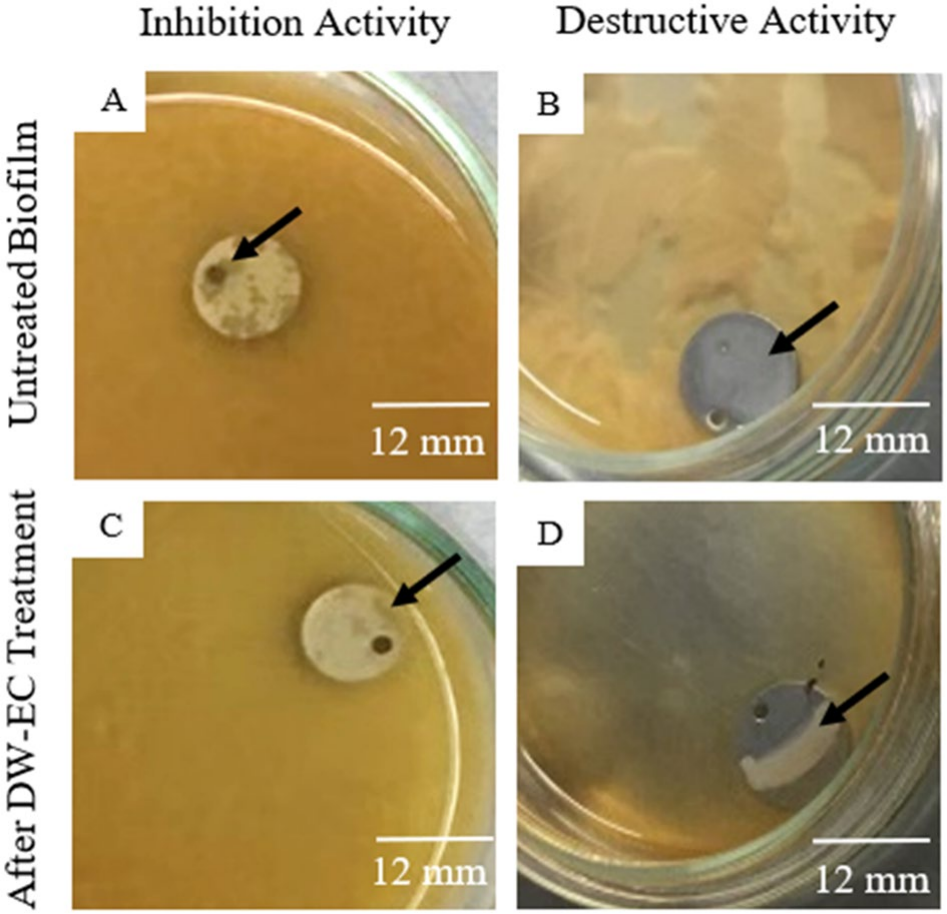
Inhibition and destructive activity of bacteriophage DW-EC against EHEC on stainless-steel: (**A**, **B**) untreated biofilm and (**C**, **D**) biofilm treated with DW-EC treatment. Black arrows indicate biofilm.

Bacteriophage DW-EC was found to inhibit and destroy foodborne pathogenic biofilms formed on stainless steel (Fig. 3). However, the inhibitory and destruction activity was lower than on the polystyrene substrate (Fig. 2).

### Microscopic visualization

**Figure 4:**
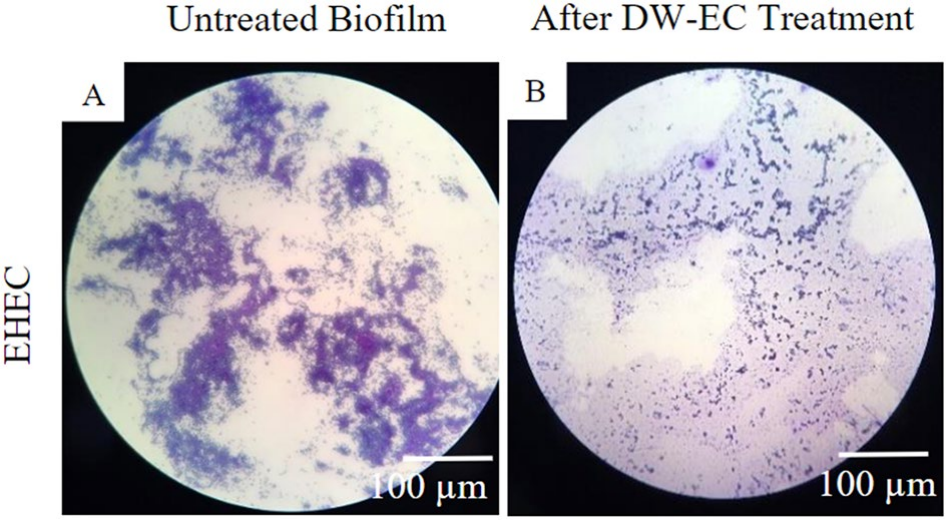
Biofilm formation inhibition of EHEC: (**A**) untreated biofilm and (**B**) biofilm formation treated with bacteriophage DW-EC. Magnification: 400**×**.

**Figure 5:**
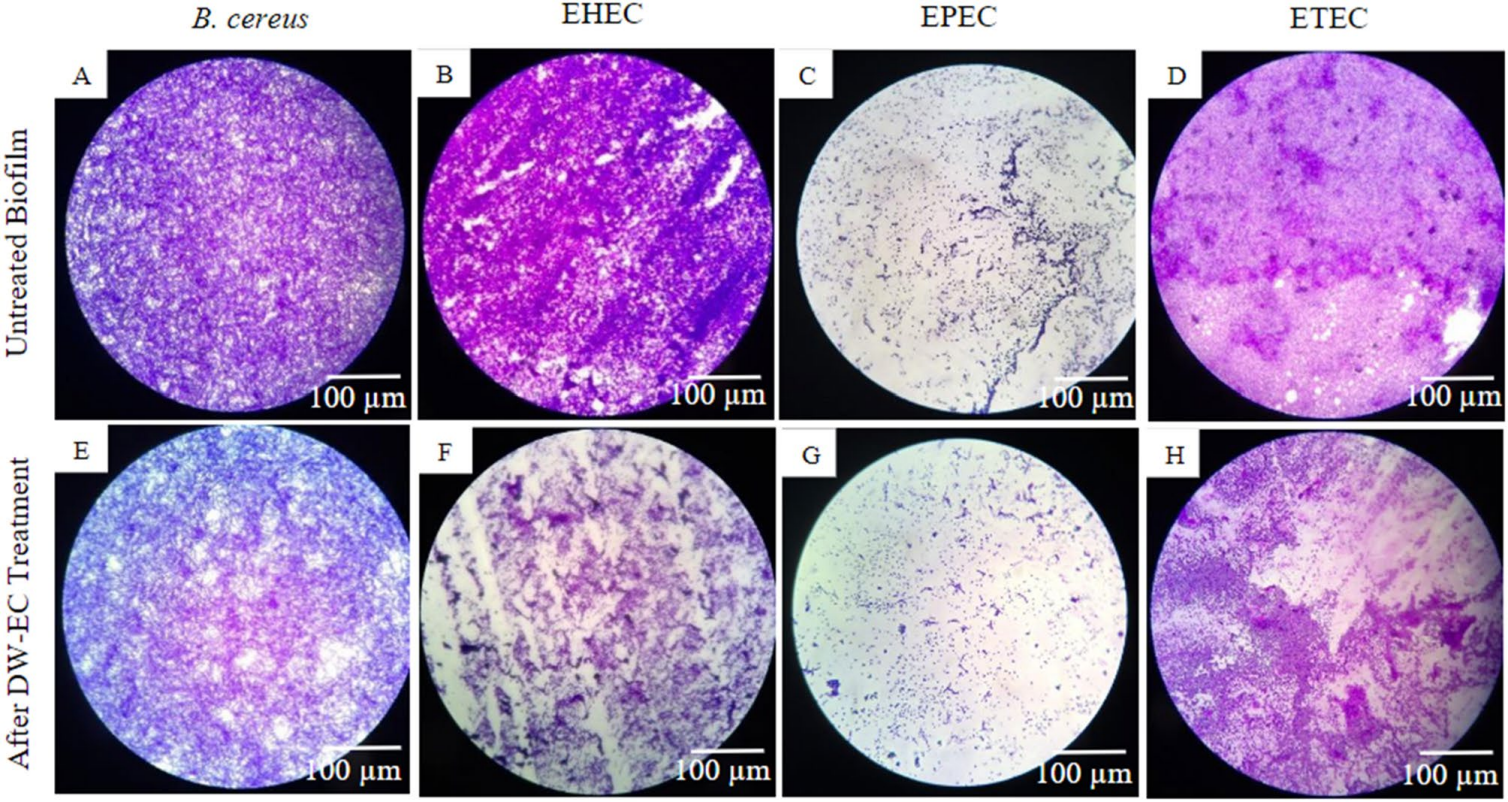
Destruction activity of bacteriophage DW-EC against biofilm formed by several pathogenic bacteria: (**A**–**D**) untreated biofilm and (**E**–**H**) biofilm treated with DW-EC. Magnification: 400×.

**Figure 6:**
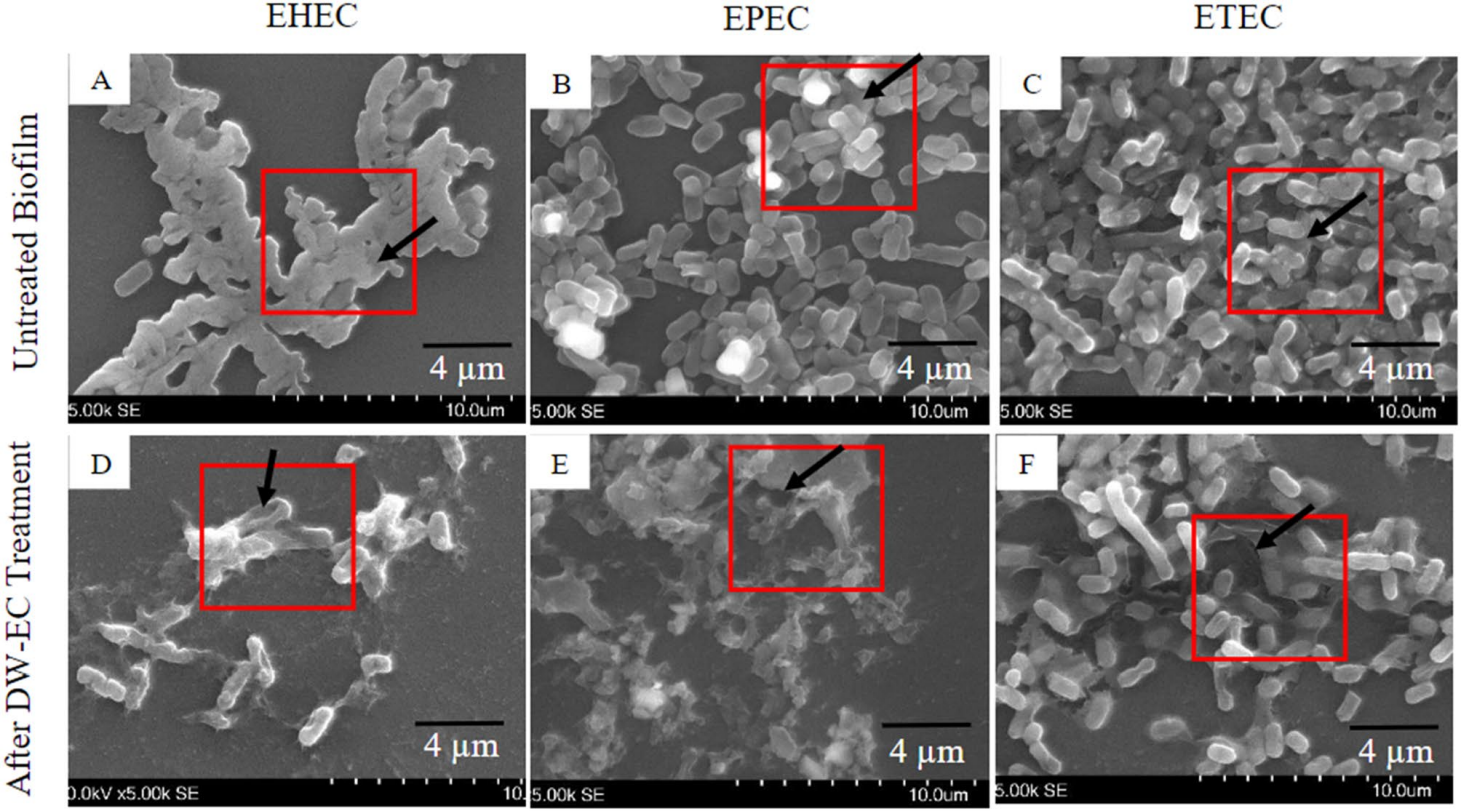
SEM of destruction activity of bacteriophage DW-EC against biofilm formed by several pathogenic bacteria: (**A**–**C**) untreated biofilm and (**D**–**F**) biofilm treated with DW-EC. Red box shows biofilm pathogens. Black arrows indicate untreated biofilm (**A**–**C**), disruption of the biofilm (**E**, **F**), and bacterial cell wall damage (**D**). Magnification: 5000×.

LM and SEM were used to observe the ability of DW-EC to destroy and inhibit the biofilm activity of pathogenic bacteria (Figs. 4, 5 and 6). Besides that, SEM was used to verify biofilm damage due to the destructive activity of DW-EC. After treatment with bacteriophage DW-EC, the biofilm structures formed by EHEC, EPEC, and ETEC were inhibited and disrupted, although the phage had no significant effect on *B. cereus* biofilms.

## Discussions

Bacteriophages are classified by their morphology and nucleic acid type. Transmission electron microscopy (TEM) is used to reveal the morphology of bacteriophages for further classification into tailed and non-tailed phages. A previous study revealed that bacteriophage DW-EC was classified into the *Myoviridae* family, which has larger head sizes with a long, rigid, contractile tail, and head and tail lengths of 75 nm and 85 nm, respectively^4,5^.

Bacteriophage DW-EC showed high specificity against the foodborne pathogenic bacteria EHEC, EPEC, and ETEC. The specificity of phage infection of bacteria depends on the adsorption capacity of the phage and the recognition of bacterial receptors during the infection process. Each cell can have different receptor locations depending on the phage and host species. In Gram-negative bacteria, a receptor can be flagella, pili, capsules, lipopolysaccharides (LPS), and surface proteins. The different modes of bacteriophage DW-EC attachment to a receptor on pathogenic *E. coli* can be caused by differences in the structure or composition of the O antigen in LPS^6,7^.

The infection process begins with the introduction of the phage through reversible adsorption to the first receptor (usually a sugar motif on the surface glycan) on the host cell, followed by irreversible binding to the host cell surface receptor. The second binding will be followed by the injection of the phage DNA into the host. This receptor will be recognized by receptor binding proteins (RBPs) on the phage tail fiber. Gram-negative bacteria are known to use O-antigen, and enterobacterial common antigen (ECA), known as primary receptors on Gram-negative bacteria. Meanwhile, secondary receptors for Gram-negative bacteria include LPS sugars or membrane proteins^8^.

Phylogenetic tree analysis was used in a previous study to determine the relationship between bacteriophage DW-EC phage tail fiber to other phages. The DW-EC putative phage tail fiber protein was found to be closely related to *Escherichia* phage ukendt putative tail fiber protein^4^. *Escherichia* phage ukendt recognizes the glycan surface and ECA as the first receptor and the first glucose of the outer membrane LPS as the second receptor. Based on these results, we assume that bacteriophage DW-EC has the same recognition receptor as *Escherichia* phage ukendt^8^.

We showed via the host spectrum determination assay that the bacteriophage DW-EC had high specificity against EHEC, EPEC, and ETEC with EOP values above 0.7 (Table 1). EOP values are classified into 4 categories: (1) 0.5–1 as high efficiency, (2) 0.2–0.5 as moderate efficiency, (3) 0.001–0.2 as low efficiency, and (4) 0.00 as inefficient^9^. Bacteriophage DW-EC had high efficiency against EHEC, EPEC, and ETEC but was ineffective against *B. cereus* biofilms. We propose that bacteriophage DW-EC has a high specificity for pathogenic *E. coli* and can be used to control the growth of these three pathogenic *E. coli* types.

EHEC, EPEC, ETEC, and *B. cereus* can grow on food processing equipment and packaging surfaces such as polystyrene and stainless steel. Polystyrene is often used for packaging meat and fruit. Polystyrene is a strong, inexpensive, and easy-to-produce material that is often used in the food industry^10^. Stainless steel is often used in food processing equipment^11^. We showed that specific foodborne pathogens could produce biofilms on polystyrene and stainless steel.

Biofilm formation is a complex but well-regulated process that can be categorized into five stages, namely: (1) reversible attachment, (2) irreversible attachment, (3) microcolony formation, (4) maturation, and (5) dispersal. Biofilm formation begins with the attachment of planktonic bacterial flagella to biotic or abiotic surfaces and cell proliferation. Several factors are involved in the initial adhesion, including polarity, London-van der Waals forces, and hydrophobic interactions. This interaction allows bacterial attachment to the surface to be more easily removed. Irreversible attachment makes bacterial attachment to the surface more stable to form a biofilm. In the next stage, microbes multiply and form several layered cells cluster, starting from microcolonies to macro-colonies, and eventually, these macro-colonies are enveloped by EPS which will develop into mature biofilms. Dispersal biofilm is the stage of release of planktonic bacteria caused by local damage to the biofilm^12^.

For inhibitory activity, pathogenic bacteria are treated with phages to prevent them from forming or inhibiting biofilm growth (0-day old biofilm). For destructive activity, pathogenic bacteria will form biofilm and be treated with bacteriophage to disrupt the mature biofilm that has already been formed (1-day old biofilm). Based on these activities, DW-E phages target the stages of microcolony formation and biofilm maturation. DW-EC can inhibit biofilm formation by lysing cells from within using lysine and putative T4-like lysozyme, attacking each cell individually, lyses cells, and inhibits proliferation, thereby preventing bacteria from reaching the required density to produce EPS. Moreover, mature biofilms are destroyed when depolymerase enzymes degrades bacterial polysaccharides^4,13^.

The inhibitory and destroying activity of biofilm was lower than that of polystyrene (Fig. 2). Moreover, weak to moderate biofilm formation was observed on stainless steel, but on polystyrene, strong biofilm formation occurred. These results confirm the findings of previous studies in which *E. coli* strains O113, O145, O91, O157, and O103 formed strong or moderate biofilms on polystyrene^14^. The surface type can affect the binding strength between the bacteria and the substrate. The biofilm formed on polystyrene is stronger and less easily detached than stainless steel. The stainless surface is hydrophilic and has a negative charge at neutral pH. The surfaces of bacterial cells also have a negative charge, making it difficult for bacteria to colonize these surfaces^15^.

Biofilm growth was seen not covering the entire 96-well base plate (Fig. 1), similar to a previous study in which the biofilm was observed to coagulate in the center and along the 96-well plate wall^15^. On the other hand, more biofilm was formed and suspend on the media than on stainless steel (Fig. 3). The biofilm formed in the 96-well plate covers a small growth area, whereas, in a petri dish, the growth area may be larger.

Although DW-EC was ineffective against *B. cereus* cells, the *B. cereus* biofilm was still affected, with inhibition and destruction values of 9.38% and 13.02%, respectively. Phages have tail fibers that function to recognize host receptors. When the host receptor cannot be accessed or recognized by the phage RBP, the phage cannot infect the host^16^. *Bacillus cereus* does not have a receptor that the tail fiber of bacteriophage DW-EC can recognize, but the depolymerase enzyme from the phage can disrupt and damage the biofilm^3^.

In contrast, bacteriophage DW-EC can attack EHEC, EPEC, and ETEC because these bacteria have recognition receptors for the bacteriophage. The bacteriophage disperses the chemical components of the biofilm matrix, especially EPS. Phages penetrate the biofilm and damage the biofilm structure with or without killing the host cell^3^. Previous research found that phage FP43 was active against planktonic bacterial cells and inhibited and degraded a biofilm of *E*. *coli* O157:H7 and O19:H–. This phage decreased the formation of biofilms up to 82.4%^17^. DW-EC is also known to inhibit the growth of bacterial cells so that they cannot reach the required density to form biofilms. The depolymerase enzyme produced in the late lytic phase can damage the biofilm matrix. Bacteriophages isolated from cow manure were found to inhibit MRSA and *E. coli* biofilm formation on polystyrene^18^. In addition, phage *E*. *coli* IV prevented and degraded biofilm growth up to 40%^19^.

Comparison of the inhibitory and destructive activity of phage DW-EC with phage EcoM017 which both attacked *E. coli* on the polystyrene surface, showed that the higher the concentration of phage given, the more damage and inhibition that occurred. Phage EcoM017 with a titer of 10^9^ PFU/mL inhibited formation and damage biofilm at 90.00% and 87.5%, respectively. These results were higher than the phage DW-EC with a titer of 10^7^ PFU/mL which had the highest inhibitory and destruction activities at 44.06% and 48.13%, respectively^20^. Furthermore, a recent report showed phage FP43 with a titer of 10^10^ PFU/mL inhibited the formation of biofilm E. coli O157:H7 and O19:H– up to 82.4%^17^. The concentration of phage titer may affect the effectiveness of phage in reducing their activity to form and destroy biofilms. Phage titers at high concentrations tend to have higher metabolic activity than biofilms^20^.

Bacteriophage DW-EC showed the highest inhibitory and destructive activity against pathogenic *E. coli*. The results were also visible via LM, as the biofilm formed by pathogenic *E. coli* after treatment with DW-EC showed disrupted biofilm structure compared to control (Figs. 4, 5).

Furthermore, determination of biofilm control and visualization via SEM also verified that the biofilm structure was disturbed and destroyed after bacteriophage DW-EC treatment (Fig. 6). Biofilms not treated with bacteriophage DW-EC showed a rough, dense, and large surface structure. In addition, bacteriophage DW-EC was found to lyse the bacterial cell wall, seen from the irregular shape of the cell. This observation indicates that adding DW-EC can decrease the number of bacterial cells and degrade their biofilm matrix^4,21^.

In our previous molecular analysis study, we found that bacteriophage DW-EC had a putative T4-like lysozyme associated with cell lysis. A putative T4-like lysozyme is a hydrolytic enzyme that can break the 1,4-glycosidic bond with N-acetylglucosamine, resulting in cell wall perforations and eventual host cell lysis^4^. In addition, this phage produces endolysin enzymes that can damage the peptidoglycan in the bacterial cell wall, also resulting in lysis^3^. Bacteriophage DW-EC lacks integrase and excisionase genes associated with the lysogenic phage stage, indicating that DW-EC phages are lytic^4^, avoiding the disadvantage of gene transfer which can prevent the transmission of antibiotic resistance genes and other virulence-associated genes that make bacterial pathogens more difficult to eradicate^22^.

In the lytic stage, bacteriophage DW-EC will enter the *E. coli* cell and utilize its genetic material and transcription and translation processes to produce as many virions as possible. In the late stage, the phage will release virions from the host cell, which will induce the release of the enzyme holin, which endolysin can use to degrade bacterial peptidoglycan. Low peptidoglycan content can cause bacteria to undergo hypotonic lysis^3^.

As the bacteriophage DW-EC was more effective at destroying biofilms, the bacteriophage can be used to decrease the number of bacteria growing on industrial food processing equipment contaminated with EHEC, EPEC, and ETEC matrix biofilms. Moreover, bacteriophage DW-EC can be used to prevent biofilm formation on industrial equipment, reducing the possibility of food product contamination by foodborne pathogens.

## Methods

### Bacterial strains

In this study we used EHEC, EPEC, ETEC from United State Naval Medical Research Unit Two (US Namru-2), USA and *B. cereus* 10,876 from American Type Culture Collection [ATCC], USA. Bacteria were grown in Luria–Bertani (LB) broth and incubated to mid-log phase at 37 °C and 120 rpm for 6–8 h. Bacteria cultures were measured at a wavelength of 600 nm using a spectrophotometer and diluted until their absorbance reached OD_600_ = 0.132/10^8^ CFU/mL and used as working cultures^4^.

### Chemical and reagents

In this study we used chemical and reagent such a CaCl_2_·2H_2_O, MgSO_4_·7H_2_O, acetic acid (glacial) 100% anhydrous for analysis, glutaraldehyde, dimethyl sulfoxide (DMSO) for analysis, ethanol absolute for analysis, NaCl, and Tris-Cl obtained from Merck, Germany. Reagents and other chemicals used as a Ringer solution (Oxoid, Great Britain), 5.25% of hypochlorite (SC Johnson, Indonesia), crystal violet, and powdered gelatine (Sigma-Aldrich, USA).

### Bacteriophage refreshement and purification

We used phage DW-EC, isolated in a previous study from *dawet*, a traditional Indonesian ready-to-eat (RTE) food, using the ETEC strain as the bacterial host. Successful phage refreshment was determined using the double overlay agar method. Bacteriophage DW-EC was refreshed by mixing 200 µL of phage with 200 µL of mid-log phase ETEC, 50 µL of 10 mM CaCl_2_, and 10 µL of 10 mM MgSO_4_ and the mixture was incubated for 20 min at 28 °C. The mixture was mixed and vortexed with 5 mL of molten LB agar (0.6% w/v agar) and poured onto LB agar (2% w/v agar), which was incubated overnight at 37 °C, and plaque formation was observed^23–25^.

For phage purification, we obtained a single plaque from the previous step collected using a sterile tip and suspended the plaque in LB broth with 200 µL of mid-log phase ETEC, 50 µL of 10 mM CaCl_2_, and 5 µL of 10 mM MgSO_4_. The mixture was incubated at 37 °C and 120 rpm overnight. It was centrifuged at 7000 rpm for 15 min to separate the bacteriophage from host cells and filtered using a 0.22 µm pore size membrane filter (HIMEDIA, India) to obtain a bacteriophage lysate. The bacteriophage lysate was stored in Ringer’s solution (1:1 v/v) at 4 °C and used as a working solution^25,26^.

#### Bacteriophage titer determination

The bacteriophage titer was determined using the double agar overlay test method. The ETEC strain was used as the bacteria host for phage DW-EC. The phage lysate was serially diluted in ten-fold dilutions using sodium of magnesium (SM) buffer using Sodium of Magnesium (SM) buffer [0.1 M NaCl, 8 mM MgSO_4_.7H_2_O, 50 mM Tris-Cl pH 7.5, 0.01% gelatine (w/v)]. Dilutions from 10^−4^ to 10^−8^ were used to determine titer. For the double agar overlay test, we followed the steps included in the bacteriophage refreshment^23–25^. Visible plaques were counted at the dilution in which 30 and 300 plaques were observed. The bacteriophage titer of the stock solution was determined as plaques per millimeter (PFU/mL)^27^. The titer determination of phage was conducted in triplicate.

### Host spectrum determination and efficiency of plating (EOP)

Bacteriophage host range and efficiency of plating (EOP) were determined using the double overlay agar method as previously described^23–25^. The pathogenic bacteria used in the host determination assay were *B. cereus*, EHEC, EPEC, and ETEC. ETEC was used as the host cell, and other pathogenic bacteria were used as other targets. The phage lysate was serially tenfold diluted (10^–4^ to 10^–8^) in SM buffer. The presence of the phage was confirmed by plaque formation after overnight incubation at 37 °C. If the 10^–4^ dilution did not produce plaques, it was necessary to use a lower dilution to verify that the EOP was lower than 0.001. Below is the equation to calculate the EOP value^27,28^.

$$ EOP = \frac{{Concentration \left( {\frac{PFU}{{mL}}} \right)t\arg et\, bacteria = \frac{Number \,of\, plaque}{{delution \times volume\, of\, phage}}}}{{Concentration \left( {\frac{PFU}{{mL}}} \right) host\, bacteria = \frac{Number\, of\, plaque}{{Delution \times volume\, of\, phage}}}} $$

### Antibiofilm activity of DW-EC on polystyrene

The colorimetric method was used to enumerate the reduction in bacterial biofilms. Each bacterial strain was cultured into brain heart infusion broth (BHIB; Oxoid, Great Britain) with 2% glucose (w/v) and incubated at 37 °C and 120 rpm overnight. Bacterial cultures were diluted to obtain an absorbance of OD_600_ = 0.132. For biofilm destruction, 100 μL of each bacterial strain (10^5^ CFU/mL) was added to 96-well plates and incubated at 37 °C for 24 h for mature biofilm formation and attachment in the well. After incubation, 100 μL of phage lysate with a multiplicity of infection (MOI) of 100 was added^24,25^. For biofilm inhibition, 100 μL of each bacterial strain (10^5^ CFU/mL) and 100 µL of phage lysate with an MOI of 100 were added to 96-well plates and incubated at 37 °C for 24 h^29^.

After incubation, the nonadherent bacteria were removed and washed twice using sterile water and air-dried for 30 min. Subsequently, 200 µL of 0.4% crystal violet was added, and the plates were incubated at room temperature for 30 min. The wells were rinsed three times with sterile water to remove stains, and crystal violet was solubilized in 200 µL ethanol. The dye absorbance at 595 nm was measured using the microplate reader. The negative control was BHIB and the positive control was bacteria culture without the phage^29^.

### Antibiofilm activity of DW-EC on stainless steel coupons

Type-201 stainless steel coupons (1.2 mm × 12 mm) were used to assess biofilm formation on specific surfaces obtained from local market. For pre-treatment, coupons were sterilized using 10% bleach (0.5% hypochlorite) for 24 h. The coupons were rinsed three times with sterile water and air-dried in a biosafety cabinet. The coupons were soaked with 70% ethanol, air-dried at room temperature for 5 min, and autoclaved at 121 °C for 15 min^15^.

Each bacterial strain was cultured into BHIB with 2% glucose (w/v) and incubated overnight at 37 °C and 120 rpm. For biofilm destruction, 2 mL of each bacterial strain (10^5^ CFU/ mL) was added to a Petri dish and incubated at 37 °C for 24 h for mature biofilm formation and attachment in the well. After incubation, 2 mL of phage lysate with an MOI of 100 was added to the Petri dish and incubated at 37 °C for 24 h. For biofilm inhibition, 2 mL of each bacterial strain (10^5^ CFU/mL) and 2 mL of phage lysate with an MOI of 100 were added and incubated at 37 °C for 24 h. After incubation, the nonadherent bacteria were removed and washed three times using sterile water and air-dried for 2 min, then 3 mL of 0.4% crystal violet was added and incubated at room temperature for 15 min. Then, the well was rinsed three times with sterile water to remove stains, and crystal violet was dissolved using 33% glacial acetic acid (Sigma-Aldrich). Dye absorbance at 590 nm was measured using the microplate reader^15^.

### Microscopic visualization

As a preliminary detection method, the structure of biofilm was visualized by using a light microscope (LM)^30^ and verified using a scanning electron microscope (SEM)^15^. The samples demonstrating the highest biofilm inhibition and destruction activity were analyzed. For the positive control, a cover glass was inoculated with 100 μL of bacterial culture and 100 μL of 1% DMSO. For biofilm destruction, approximately 4 mL of each pathogen (10^5^ CFU/mL) was added onto a cover glass and incubated at 37 °C overnight for mature biofilm formation and attachment in the well. After incubation, the cover glass was transferred into a new Petri dish, 100 μL of phage lysate with an MOI of 100 was added, and the glass re-incubated. For biofilm inhibition, 100 μL of each bacterial strain and 100 μL of phage lysate with an MOI of 100 were added to the cover glass and incubated at 37 °C overnight.

For LM observation, the nonadherent bacteria were rinsed with water and air-dried for 2 min, then 1 mL of 0.4% crystal violet was added, and the glass was incubated at room temperature for 15 min. The stained biofilm was rinsed with sterile water to remove stains and observed at a magnification of 400×^30^.

For observation by SEM, the cover glass was fixed using 2.5% glutaraldehyde (v/v) and incubated overnight 4 °C. Samples were dehydrated with serial ethanol (30% v/v for 15 min, 50% v/v for 15 min, 70% v/v for 15 min, 96% v/v for 15 min, and 100% v/v for 15 min). The cover glass was dried at 37 °C for 10 min^15^.

### Statistical analysis

Destruction and inhibition data were analyzed using IBM^®^SPSS^®^ for Windows (version 26) with Tukey’s b post-hoc test with the level of difference defined at *P* ≤ 0.05^31^. Values of *P* below 0.05 were considered statistically significant. Different letters in each column indicate a significant difference between each sample. All data were presented in %, except titer and EOP, which were presented as means ± standard error.

## Conclusions

Bacteriophage DW-EC isolated from dawet using ETEC as a host cell showed an effective attack on EHEC, EPEC, and ETEC as indicate by the EOP value above 0.07. Phage DW-EC produces several enzymes that can be used as anti-biofilms. Depolymerase enzymes are produced to damage the EPS layer, causing disruption and destroying the biofilm. Besides that, lisin and putative T4-like lysozyme were perforating the cell wall and causing lysing of the host cells. This study aimed to determine the ability of DW-EC phages to inhibit and destroy biofilms produced by several foodborne pathogens. The results showed that DW-EC had potential activity to control bacterial growth and inhibit and disrupt biofilms formed by the foodborne pathogenic bacteria used in this study. Phage DW-EC can reduce bacterial populations and control biofilms on polystyrene and stainless steel. Bacteriophage DW-EC showed inhibitory and destruction activity of biofilm on polystyrene of EHEC (44.06%; 48.13%), EPEC (39.28%; 44.64%), ETEC (24.19%; 39.17%), and *B. cereus* (14.34%; 16.13%). Meanwhile, the inhibitory and destruction activity of biofilm on stainless-steel of EHEC (27.91%; 38.24%), EPEC (22.89%; 38.46%), ETEC (16.67%; 27.00%), and *B. cereus* (9.38%; 13.02%). The structures formed by EHEC, EPEC, and ETEC biofilms after treatment with DW-EC were inhibited and disrupted compared to the control. Phage DW-EC was confirmed to be effective in controlling biofilms of EHEC, EPEC, and ETEC by SEM visualization. Bacteriophage DW-EC is promising as an anti-biofilm agent for application in food industry environments, especially to treat biofilms on polystyrene packaging and stainless-steel equipment.

## Data Availability

All data generated or analyzed during this study are included in this published article.

## References

[1] Sadekuzzaman M, Yang S, Mizan MdFR, Ha S-D (2017). Reduction of *Escherichia coli* O157:H7 in biofilms using bacteriophage BPECO 19: Phage control *E. coli* O157:H7 in biofilm. J. Food Sci..

[2] Galie S, Garcia-Gutierrez C, Miguelez EM, Villar CJ, Lombo F (2018). Biofilms in the food industry: Health aspects and control methods. Front. Microbiol..

[3] Kifelew LG, Mitchell JG, Speck P (2019). Mini-review: Efficacy of lytic bacteriophages on multispesies biofilm. J. Bioadhesion Biofilm Res..

[4] Dewanggana MS (2022). Isolation, characterization, molecular analysis and application of bacteriophage DW-EC to control Enterotoxigenic *Escherichia coli* on various foods. Sci. Rep..

[5] Ketty M. D. *Isolation and Characterization of Pathogenic Escherichia coli Bacteriophage from Ready-to-eat Foods [Thesis]* (Unika Atma Jaya, 2019).

[6] Letellier, L., Plancon, L., Boulanger, P. *Transfer of DNA from Phage to Host. Bacteriophage: Genetics and Molecular Biology* (eds. Mc, G.S. & van, S.D.) (Caister Academic 2007).

[7] Arivo D, Rusmana I, Budiarti S (2016). Isolation and characterization of EPEC phage from domestic waste in Indonesia. Malays. J. Microbiol..

[8] Maffei E (2021). Systematic exploration of *Escherichia coli* phage-host interactions with the BASEL phage collection. PLoS Biol..

[9] Islam MS (2019). Application of a phage cocktail for control of *Salmonella* in foods and reducing biofilms. Viruses.

[10] Angarano V (2020). A reproducible method for growing biofilms on polystyrene surfaces: biomass and bacterial viability evolution of *Pseudomonas fluorescens* and *Staphylococcus epidermidis*. Appl. Sci..

[11] Paz-Méndez AM (2017). Effect of food residues in biofilm formation on stainless steel and polystyrene surfaces by *Salmonella enterica* strains isolated from poultry house. Foods.

[12] Liu S, Lu H, Zhang S, Shi Y, Chen Q (2022). Phages against pathogenic bacterial biofilms and biofilm-based infections: A review. Pharmaceutics..

[13] Tian F, Li J, Nazir A, Tong Y (2021). Bacteriophage - A promising alternative measure for bacterial biofilm control. Infect. Drug Resist..

[14] Wang J (2016). Biofilm formation virulence gene profiles, and antimicrobial resistance of nine serogroups of Non-O157 Shiga Toxin-Producing *Escherichia coli*. Foodborne Pathog. Dis..

[15] Lorenzo F, Sanz-Puig M, Bertó R, Orihuel E (2020). Assessment of performance of two rapid methods for on-site control of microbial and biofilm contamination. Appl. Sci..

[16] Silva JB, Storms Z, Sauvageau D (2016). Host receptors for bacteriophage adsorption. FEMS Microbiol. Lett..

[17] Zhang Y (2020). Effect of bacteriophage on inhibition and removal of mixed biofilm of enterohemorrhagic *Escherichia coli* O157:H7 and O19:H-. LWT..

[18] Kumari N, Patoli BB, Patoli AA, Jabeen S (2020). Biocontrol of MRSA and *E. coli* using bacteriophage from cow manure. Adv. Life Sci..

[19] Yulinery, T., Triana, E., Suharna, N., Nurhidayat, N. Isolation and anti-*Escherichia coli* biofilm activity of lytic bacteriophage isolated from water environment in vitro. In *IOP Conference Series: Earth and Environmental Science* 308 (2019).

[20] Ribeiro C (2018). Bacteriophage isolated from sewage eliminates and prevent the establishment of *Escherichia coli* Biofilm. Adv. Pharm. Bull..

[21] Gómez-Gonzáles JP (2021). Efficiacy of novel bacteriophage against *Escherichia coli* biofilm on stainless steel. Antibiotics.

[22] Borodovich T, Shkoporov AN, Ross RP, Hill C (2022). Phage-mediated horizontal gene transfer and its implications for the human gut microbiome. Gastroenterol. Rep..

[23] Bandara N, Jo J, Ryu S, Kim K-P (2012). Bacteriophages BCP1-1 and BCP8-2 require divalent cations for efficient control of *Bacillus cereus* in fermented foods. Food Microbiol..

[24] Thung TY (2017). Use of a lytic bacteriophage to control *Salmonella Enteritidis* in retail food. LWT.

[25] Rai S, Tyagi A, Kumar BN, Singh N (2020). Optimization of plaque forming conditions for an *Aeromonas hydrophila* lytic bacteriophage. Int. J. Curr. Microbiol. Appl. Sci..

[26] Snyder AB, Perry JJ, Yousef AE (2016). Developing and optimizing bacteriophage treatment to control enterohemorrhagic *Escherichia coli* on fresh produce. Int. J. Food Microbiol..

[27] Khan Mirzaei M, Nilsson AS (2015). Isolation of phages for phage therapy: A comparison of spot tests and efficiency of plating analyses for determination of host range and efficacy. PLoS ONE.

[28] Jurczak-Kurek A (2016). Biodiversity of bacteriophages: Morphological and biological properties of a large group of phages isolated from urban sewage. Sci. Rep..

[29] Mulya E, Waturangi DE (2021). Screening and quantification of-anti quorum sensing and antibiofilm activity of *Actinomycetes* isolates against food spoilage biofilm-forming bacteria. BMC Microbiol..

[30] Thenmozhi R, Nithyanand P, Rathna J, Pandian SK (2009). Antibiofilm activity of coral-associated bacteria against different clinical M serotypes of *Streptococcus pyogenes*. FEMS Immunol. Med. Microbiol..

[31] Lukman C, Yonathan C, Magdalena S, Waturangi DE (2020). Isolation and characterization of pathogenic *Escherichia coli* bacteriophages from chicken and beef offal. BMC Res. Notes.

